# Long-Lasting T Cell Responses in BNT162b2 COVID-19 mRNA Vaccinees and COVID-19 Convalescent Patients

**DOI:** 10.3389/fimmu.2022.869990

**Published:** 2022-04-22

**Authors:** Antti Hurme, Pinja Jalkanen, Jemna Heroum, Oona Liedes, Saimi Vara, Merit Melin, Johanna Teräsjärvi, Qiushui He, Sakari Pöysti, Arno Hänninen, Jarmo Oksi, Tytti Vuorinen, Anu Kantele, Paula A. Tähtinen, Lauri Ivaska, Laura Kakkola, Johanna Lempainen, Ilkka Julkunen

**Affiliations:** ^1^ Institute of Biomedicine, University of Turku, Turku, Finland; ^2^ Department of Infectious Diseases, Turku University Hospital and University of Turku, Turku, Finland; ^3^ Department of Health Security, Finnish Institute for Health and Welfare, Helsinki, Finland; ^4^ Clinical Microbiology, Turku University Hospital, Turku, Finland; ^5^ Meilahti Vaccine Research Center, MeVac, Department of Infectious Diseases, Helsinki University Hospital and University of Helsinki, Helsinki, Finland; ^6^ Department of Paediatrics and Adolescent Medicine, Turku University Hospital and University of Turku, Turku, Finland

**Keywords:** Covid-19, BNT162b2, vaccine, T cell mediated immunity, humoral immunity

## Abstract

The emergence of novel variants of the severe acute respiratory syndrome coronavirus 2 (SARS-CoV-2) has made it more difficult to prevent the virus from spreading despite available vaccines. Reports of breakthrough infections and decreased capacity of antibodies to neutralize variants raise the question whether current vaccines can still protect against COVID-19 disease. We studied the dynamics and persistence of T cell responses using activation induced marker (AIM) assay and Th1 type cytokine production in peripheral blood mononuclear cells obtained from BNT162b2 COVID-19 mRNA vaccinated health care workers and COVID-19 patients. We demonstrate that equally high T cell responses following vaccination and infection persist at least for 6 months against Alpha, Beta, Gamma, and Delta variants despite the decline in antibody levels.

## Introduction

Severe acute respiratory syndrome coronavirus 2 (SARS-CoV-2) causes coronavirus disease 2019 (COVID-19) – an acute respiratory infection with a highly variable disease outcome. Extensive research efforts have enabled a rapid development of different types of vaccines and their implementation in clinical practice (1). However, the emergence of different SARS-CoV-2 variants of concern (VOCs), such as the Beta (B.1.351) and Delta (B.1.617.2) variants as well as the most recent variant, Omicron (B.1.1.529), has raised concerns about breakthrough infections, since these variants carry mutations in the spike protein that is the target of vaccine-induced immunity. Significant reduction in the capacity of circulating antibodies induced by vaccination or natural infection, to cross-neutralize VOCs have been reported (2–4). Fortunately, antibodies are not the only mediators of immune protection against COVID-19. Alongside virus-specific antibodies, T cells play an essential role in protection and recovery from an acute infection and in long-lasting immune memory. T cells recognize multiple short peptides of viral proteins that are presented by antigen-presenting cells (APCs), such as monocyte/macrophages and dendritic cells (5). Since T cell responses are often stimulated by dozens of different peptides of a target protein, the overall functionality of the T cell response is not as sensitive to mutations or antigenic variation in proteins as humoral responses.

CD8+ cytotoxic T lymphocytes (CTLs) recognize and eliminate virus-infected cells while CD4+ T helper cells mainly coordinate and enhance CTL responses and stimulate antibody production by B cells (6, 7). SARS-CoV-2 infection seems to stimulate robust memory CD4+ and CD8+ T cell responses which may provide long-lasting immunity against reinfections (8–10). Here, we have analyzed the longevity of SARS-CoV-2 spike-specific humoral and cell-mediated immunity in a cohort of BNT162b2 vaccinated health care workers (n=23) and COVID-19 patients (n=15). We show that even if SARS-CoV-2 spike protein-specific antibody responses decline relatively rapidly after COVID-19 vaccination, the cell-mediated immunity is to a great extent retained and remains quite insensitive to the antigenic variation in the viral spike glycoprotein.

## Materials and Methods

### Study Participants

Health care workers (HCWs, n=23) vaccinated twice with mRNA-based SARS-CoV-2 vaccine BNT162b2 (BioNTech-Pfizer) in a three-week interval were selected from a larger cohort of vaccinated individuals of HCWs from Turku University Hospital. In addition, COVID-19 patients with a PCR-confirmed SARS-CoV-2 infection (n=15) from Turku University Hospital and non-vaccinated people with no previous SARS-CoV-2 infection or COVID-19 vaccination (n=13) were included in the study. Sera and peripheral blood mononuclear cells (PBMCs) from the vaccinees were collected six weeks, three months, and six months after the first vaccine dose. Convalescent sera and PBMCs were collected 18-45 days (mean 33 days) following SARS-CoV-2 infection from patients recovered from a severe or moderate COVID-19 disease.

### Ethics

Vaccines were administrated by the occupational health care and written informed consent was collected from all the study participants before collecting the samples. The Ethics Committees of the Southwest Finland health district and the Helsinki-Uusimaa health district approved the study protocols (for vaccinees ETMK 19/1801/2020 and EudraCT 2021-004419-14, and for patients and controls HUS/1238/2020 and EudraCT 2021-004016-26).

### PBMC Isolation

Peripheral whole blood was collected into lithium-heparin vials and PBMCs were isolated using Ficoll-Paque PLUS (GE Healthcare) density gradient centrifugation according to the manufacturer’s instructions. After isolation, the PBMCs were counted, and cell viability was assessed with trypan blue dye (BioRad) with TC20 automated cell counter (BioRad). Isolated PBMCs were suspended to 5-15 x 10^6^ cells/mL in freezing medium containing 10% DMSO and 10% human AB serum (Sigma-Aldrich) and gradually frozen to -135°C until further use.

### Cell Culture and Stimulations

Cryopreserved PBMCs were rapidly thawed and washed with culture media RPMI (Gibco) supplemented with 10% human AB serum, 2mM L-glutamine, and penicillin/streptomycin). PBMCs were rested in a concentration of 5x10^6^ cells/ml for 8h at +37°C and 5% CO_2_. After resting, PBMCs were pelleted and resuspended into fresh culture media and counted with TC20 cell counter.

To activate the SARS-CoV-2 specific T cells, 1x10^6^ live cells in 100 μl culture media were plated into 96 well U-bottom plate (Thermo) wells and stimulated with peptide pools spanning the whole SARS-CoV-2 spike protein (PepMiX, JPT Peptide Technologies) at a final concentration of 0.5 µg/ml in culture media for 48h at +37°C and 5% CO_2_. Peptide pools consisted of 15mers with 11mer overlap. Ten µg/ml of purified tetanus toxoid (AJ Vaccines) and 0.4% of DMSO in culture media were used as positive and negative controls, respectively. The stimulations were done in a final volume of 200 μl of cell culture media. SARS-CoV-2 peptide pools are described at **Supplementary Table 1**.

### Flow Cytometry

Stimulated PBMCs were washed once with PBS and dead cells were stained with 1:1000 diluted Zombie Green dye (BioLegend) for 15 min at room temperature in the dark. PBMCs were washed with FACS buffer (PBS containing 2% fetal bovine serum) and subsequently stained for surface markers with a cocktail of fluorochrome-labeled anti-human antibodies recognizing CD45, CD3, CD4, CD8, CD69, CD134, and CD137 (**Supplementary Table 2**). PBMCs were incubated for 30 min at +4°C in the dark, washed with FACS buffer and re-suspended in 200µl of PBS. T cell subtypes were characterized with NovoCyte Quanteon Flow Cytometer (Agilent Technologies Inc) and analyzed with NovoExpress v1.5.0 (Agilent Technologies Inc). The gating was done manually from the main cell population of DMSO stimulated samples and the same gating was used for each respective tetanus- and S-peptide pool stimulated cells.

The exclusion criteria for a sample were a lymphocyte percentage of less than 4% in the forward vs side scatter (FSC vs SSC) gating, CD3 cell count lower than 10,000; CD4 or CD8 cell counts lower than 5,000 or total events lower than 100,000. Missing CD4+ response to tetanus toxoid (stimulation index <2.0) was considered a failure of that sample to be activated and thus resulted in exclusion from the analysis.

### RT-qPCR

The expression of cellular IFN-γ, IL-2, TNF-α and IL-4 mRNAs were measured using one-step RT-qPCR protocol. Total RNA of the stimulated PBMCs was isolated using RNeasy Mini kit (Qiagen) or MagNA Pure 96 Cellular RNA Large Volume Kit in MagnaPure 96 System (Roche) according to manufacturer’s protocols. For amplification and quantitation, 5 µl of purified RNA was used in One Step PrimeScript III RT-qPCR Kit (Takara Bio Inc) with predesigned TaqMan FAM-MGB IFN-γ (Hs00989291_m1), IL-2 (Hs00174114_m1), IL-4 (Hs00174122_m1), TNF-α (Hs00174128_m1) and β-actin (Hs01060665_g1) primer/probe sets (Thermo Fisher Scientific) in Rotor-Gene Q (Qiagen). The conditions for the qRT-PCR thermal cycling were the following: One reverse transcription cycle at 55°C for 10 min and 95°C for 10 sec followed by 45 amplification cycles at 95°C for 5 sec and 58°C for 30 sec. The relative fold changes of the target genes were obtained with the 2^−ΔΔCt^ method by using β-actin Ct-values for normalization and for each participant their corresponding DMSO treated sample as the calibrator.

### Cytokine Detection in Cell Culture Supernatants Using Luminex

The levels of secreted cytokines and other molecules (IFN-γ, IL-2, TNF-α, and perforin) in the stimulated PBMC supernatants were analyzed with a 96-well plate assay using MILLIPLEX MAP Kit HCD8MAG-15K from Millipore. The fluorescence of the samples was measured with Luminex MAGPIX magnetic bead analyzer (Luminex Corporation, Austin, TX). The concentration of each cytokine was calculated from median fluorescent intensity of 7 diluted standards using a 5-parameter logistic regression. Samples that were in the linear range were given their measured concentration. Samples below the lowest standard in the linear range were given half the value of the standard which were 2.4 pg/ml for IFN-γ, 0.9 pg/ml for IL-2, 1.0 pg/ml for TNF-α, and 6.0 pg/ml for perforin. Samples over the highest standard in the linear range were given the highest value of the standard which were 5 ng/ml for IFN-γ, 7.5 ng/ml for IL-2, 2 ng/ml for TNF-α, and 50 ng/ml for perforin. Standards with a standard deviation of less than 25% for the duplicates were accepted. According to the kit manufacturer, if there were less than 35 beads in the well, the samples could not be given a reliable concentration and thus those samples were discarded from the final analysis. Samples that had a measuring result from DMSO stimulation and either from tetanus and/or SARS-CoV-2 spike peptide pool stimulation (wild type and/or Delta) or samples that had a measuring result from SARS-CoV-2 spike peptide pool stimulation with both wild type peptides and Delta peptides were included in the final analysis.

### Enzyme Immunoassay

Anti-viral antibody levels against SARS-CoV-2 spike protein subunit (S1) and nucleocapsid (N) were measured with an in-house enzyme immunoassay (EIA) as described previously (11). Briefly, N and S1 protein precoated 96-well microtiter plates were incubated with a 1:300 dilution of the serum sample in PBS supplemented with 5% swine serum (BioInd) and 0.01% Tween-20. SARS-CoV-2 specific IgG and total Ig antibodies were detected with HRP conjugated anti-human IgG (Dako A/S) and total Ig (Abcam) antibodies (1:8000 and 1:20 000 dilution, respectively). TMB One (Kementec) was used as a substrate and the absorbance values were measured at a 450 nm wavelength. Optical density (OD) values were converted to EIA units with linear interpolation between negative (marked as 0 EIA units) and positive (marked as 100 EIA units) control samples as described previously (11).

### Statistical Analysis

Data were analyzed with GraphPad Prism (version 8). T cell data are shown as stimulation indices calculated by dividing the percentage of AIM^+^ cells after SARS-CoV-2 spike peptide pool stimulation with the percentage of AIM^+^ cells after DMSO stimulation. If the percentage of AIM^+^ cells after DMSO stimulation was 0, the stimulation index was marked as the smallest value of the participant. Paired samples were tested with Wilcoxon signed-rank test when there were three or more pairs and unpaired samples were tested with Mann-Whitney U-test. All tests were two-sided and p-values <0.05 were considered statistically significant. Correlations were analyzed with the Spearman’s correlation test. All statistical tests used have also been mentioned in the figure legends.

## Results

### Optimization of PBMC Stimulation and AIM Assay

**Figure 1:**
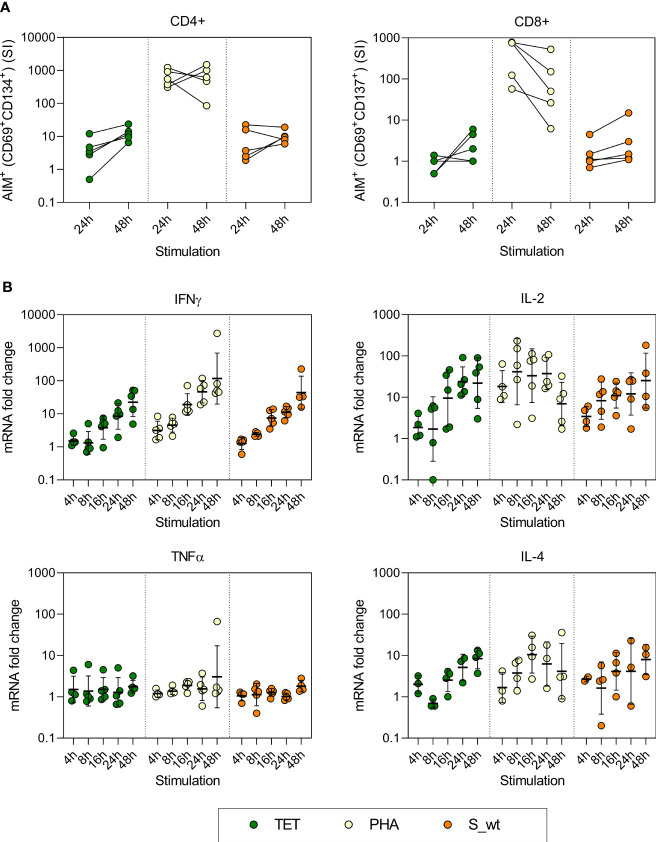
Optimization of AIM test and the kinetics of cytokine mRNA expression of stimulated PBMCs. PBMCs collected from five vaccinees were stimulated with 10 µg/ml pf tetanus toxoid (TET), 1 µg/ml PHA, and 0.5 µg/ml SARS-CoV-2 Wuhan Hu virus spike peptide pool (S_wt) for 4, 8, 16, 24, and 48h. **(A)** After 24h and 48h stimulation, specific T cell responses were measured with flow cytometry using expression of CD69 and CD134 as a marker for CD4^+^ T cell activation, and CD69 and CD137 as a marker for CD8^+^ T cell activation. Stimulation index (SI) was calculated by dividing the percentage of AIM^+^ cells after TET, PHA, or S_wt stimulation with the percentage of AIM^+^ cells after DMSO stimulation. **(B)** Cytokine mRNA expression was analyzed with RT-qPCR from total cellular RNA isolated from cells collected at different times after stimulation. RNA levels at different time points were compared to corresponding DMSO treated samples. The geometric means and standard deviations are show in the figures.

Before analyzing samples from patients and vaccinees the stimulation time was optimized. PBMCs collected 3-9 months after the second BNT162b2 vaccination from five HCWs were stimulated with 0.5 μg/ml of SARS-CoV-2 Wuhan Hu virus (wt) spike peptide pool, 10 µg/ml of tetanus toxoid, and 1 µg/ml of phytohemagglutinin (PHA) for 4, 8, 16, 24, and 48 hours. T cell activation induced markers (AIM) were analyzed with flow cytometry and the expression of cellular IFN-γ, IL-2, TNF-α, and IL-4 mRNA was measured with RT-qPCR. Longer 48-hour stimulation period increased the expression of CD4+ and CD8+ T cell activation markers in most of the participants (3 out of 5 participants for CD4+ and 4 out of 5 participants for CD8+) compared to a shorter 24-hour stimulation period (**Figure 1A**). In addition, the expression of IFN-γ and IL-2 mRNA levels gradually increased from 4h to 48h, and the expression levels were highest after tetanus toxoid and spike peptide stimulation for 48 hours (**Figure 1B**). The expression of TNF-α mRNA was not increased and the expression of IL-4 mRNA was not detectable in all the samples. PHA activated the T cells rapidly, resulting in decreasing IL-2 mRNA levels after 24h stimulation. Thus, 48h stimulation with tetanus toxoid as positive control and measurement of IFN-γ and IL-2 mRNA levels were selected for further analysis.

### Identification of SARS-CoV-2 Specific T Cell Responses After BNT162b2 Vaccination

T cell responses were studied from a cohort of 23 vaccinated health care workers and the results were compared with 15 COVID-19 patients, and 13 negative controls without a vaccination or a previous infection. Vaccinees were 26 to 60 years old (mean 39 years) and 87% (20/23) were female while COVID-19 patients were 32 to 78 years old (mean 53 years) and 40% (6/15) were female. Of the patients, 93% (14/15) were treated in the hospital, of whom 27% (4/15) required intensive care treatment (i.e. were at risk of intubation). One patient was treated at outpatient care.

**Figure 2:**
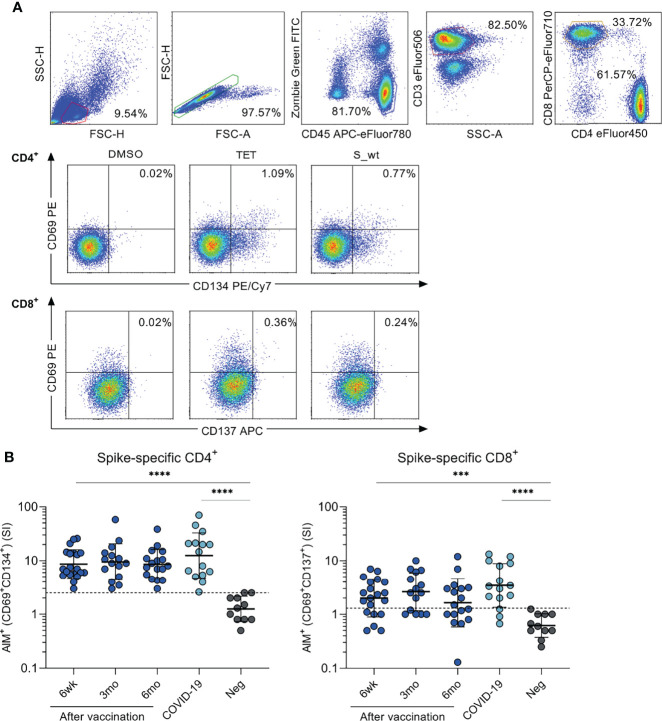
SARS-CoV-2 spike-specific CD4+ and CD8+ T cell responses after BNT162b2 vaccination. Peripheral blood mononuclear cells (PBMCs) collected from health care workers (HCWs) at different times after two doses of BNT162b2 vaccine were stimulated with wild type (wt) SARS-CoV-2 spike protein-specific peptides for 48 h. **(A)** Representative graphs from one vaccinated individual illustrating the gating strategy used for all participants in flow cytometric analysis in T cell activation induced marker (AIM) assay. Antigen-specific CD4^+^ responses were defined with the expression of CD69^+^ and CD134^+^, and CD8^+^ responses with the expression of CD69^+^ and CD137^+^. Tetanus toxoid (TET) was used as a positive control in activation experiments. **(B)** PBMCs from BNT162b2 vaccinated HCWs (n=23), convalescent COVID-19 patients (n=15), and negative non-vaccinated controls (n=13) were stimulated with SARS-CoV-2 wild type spike protein peptide pool (0.5 µg/ml) for 48h. SARS-CoV-2 spike specific responses are indicated as stimulation indices (SI) calculated by dividing the percentage of AIM^+^ (CD69^+^CD134^+^) CD4^+^ and AIM^+^ (CD69^+^CD137^+^) CD8^+^ T cells with the percentage of AIM^+^ cells after DMSO stimulation. Results are from 20, 15, and 17 samples collected 6 weeks, 3 months, and 6 months after the first vaccine dose, respectively, and 11 negative controls Comparisons between the groups were performed with the Mann-Whitney U-test and paired analysis was done with the Wilcoxon signed rank test. ***p < 0.001; ****p < 0.0001. The differences between 3wk, 3mo and 6mo sample groups were statistically not significant.

Following the activation with SARS-CoV-2 (wt) spike peptide pool, the absolute numbers, and relative frequencies of circulating CD4^+^ and CD8^+^ cell populations were determined with an 8-color flow cytometry panel with AIM assay (**Figure 2A**). SARS-CoV-2 spike-specific CD4^+^ cell responses, determined as expression of CD69^+^ and CD134^+^, were detected in all tested COVID-19 patients (n=15) and vaccinees 6 weeks (n=20), 3 months (n=15) and 6 months after the first vaccine dose (n=17) (**Figure 2B**). There was no statistically significant difference in CD4^+^ cell activation between vaccinees and COVID-19 patients. The mean stimulation indices were 8.6-9.5 for vaccinees and 12.5 for COVID-19 patients. The threshold for positive response was set based on the strongest response of the uninfected non-vaccinated control group.

CD8^+^ cell responses, determined as the expression of CD69^+^ and CD137^+^, were detected in 80% of COVID-19 patients and 70% of vaccinees six weeks after the first vaccine dose (3 weeks after the second dose), in 67% of vaccinees 3 months after the first dose, and in 53% of vaccinees 6 months after the first dose (**Figure 2B**). There was also no statistically significant difference in CD8+ cell activation between the vaccinees and COVID-19 patients. The mean stimulation indices were 1.6-2.6 for vaccinees and 3.5 for COVID-19 patients.

### T Cell Responses Against SARS-CoV-2 Variants of Concern

To assess the T cell immunity against variant strains of SARS-CoV-2, peptide pools derived from S-protein sequence of wt, Alpha, Beta, Gamma and Delta variants were used to stimulate PBMCs. Peptide pools spanning the spike proteins of wild type SARS-CoV-2 and Beta variant were used for all samples, whereas Alpha and Gamma stimulations were done for 8 and 7 vaccinees, 7 and 4 COVID-19 patients, and 6 and 3 healthy non-vaccinated controls, respectively. Delta stimulations were performed for 8 vaccinees, 6 COVID-19 patients, and 5 controls. Alpha and Gamma stimulations were performed for different participants than Delta stimulations.

**Figure 3:**
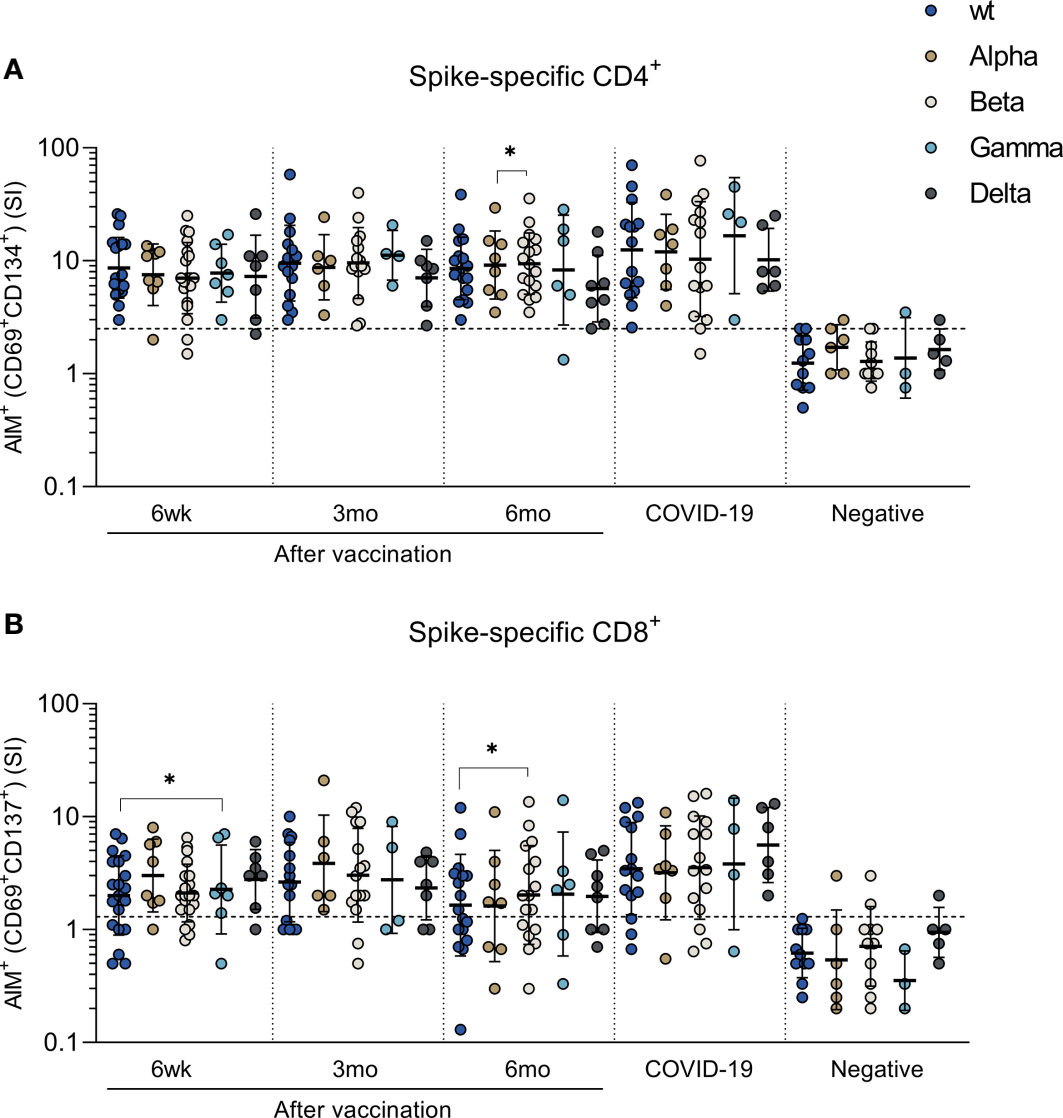
T cell responses against SARS-CoV-2 variants of concern Alpha, Beta, Gamma and Delta. PBMCs collected from HCWs after BNT162b2 vaccination, COVID-19 patients, and negative controls were treated with DMSO or stimulated with peptide pools spanning the spike protein from SARS-CoV-2 wild type (S_wt) and four variants of concern Alpha, Beta, Gamma, and Delta. Stimulation indices (SI) were calculated by dividing the percentage of AIM^+^ cells after spike peptide pool stimulation with the percentage of AIM^+^ cells after DMSO stimulation. **(A)** CD4+ and **(B)** CD8+ T cell response was analyzed with the AIM assay and antigen specific CD4^+^ responses were defined with the expression of CD69^+^ and CD134^+^, and CD8^+^ responses with the expression of CD69^+^ and CD137^+^. Paired samples were analyzed with the Wilcoxon signed rank test and samples with no data on both data points were excluded from analysis. *p < 0.05.

SARS-CoV-2 specific CD4^+^ cell responses were detected in over 71% of vaccinated individuals and in over 75% COVID 19 patients against all variants (**Figure 3A**). The mean CD4^+^ stimulation indices against variants ranged from 7 to 10 at different times after the vaccination and no statistically significant differences in CD4^+^ activation between variant peptide pools was observed except between 6-month samples against Alpha and Beta variant (p<0.01). In addition, CD8^+^ T cells were activated independent of the variant peptide pools and CD8+ T cell responses were detected in over 50% of vaccinees and in over 75% of COVID-19 patients against all variants (**Figure 3B**). Statistically significant difference was observed only between wt and Gamma 6 weeks after vaccination and wt and Beta 6 months after vaccination. COVID-19 patient-derived samples showed somewhat stronger CD4^+^ and CD8^+^ responses to peptides compared to vaccinees (**Figures 3A, B**). Importantly, there was no decrease in cell-mediated immunity against variants during the 6-month follow-up.

The cut-off for positivity was determined with wt peptide pool in negative controls. Some of the negative controls showed positive CD4+ (2 participants) or CD8+ (4 participants) responses against variant peptide pools (**Figure 3**). This weak positivity could be due to cross-reactive responses caused by previous infections with seasonal coronaviruses (12).

### Expression of Cytokine Genes and Secretion of Cytokines in SARS-CoV-2 Spike Peptide Stimulated PBMCs

**Figure 4:**
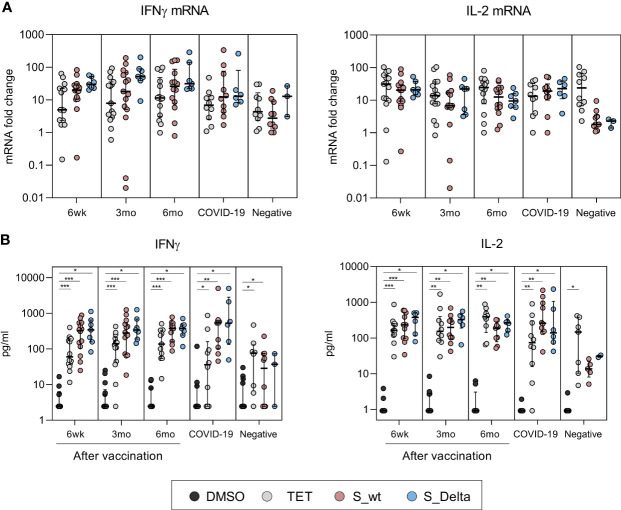
Cytokine mRNA expression in PBMCs and secretion of cytokines after stimulation with SARS-CoV-2 spike-specific peptides. Cytokine analysis was performed for PBMCs collected from BNT162b2 vaccinated HCWs 6 weeks (n=15), 3 months (n=15), and 6 months (n=14) after the first vaccine dose and from convalescent COVID-19 patients (n=10) and negative controls (n=10). PBMCs were treated with DMSO or stimulated with tetanus toxoid (10 µg/ml), SARS-CoV-2 wt or Delta S protein peptide pools (0.5 µg/ml) for 48h. Delta variant S peptide stimulation was performed to 7/15 vaccinated, 6/10 COVID-19 patients, and 3/10 negative controls. Cells and supernatants were collected, and total cellular RNA was isolated from stimulated cells. **(A)** IFN-γ and IL-2 mRNA expression was quantitated from total cellular RNA with RT-qPCR and the data is presented as fold change in comparison to DMSO treated cells. **(B)** Secreted cytokines (IFN-γ and IL-2) were analyzed with multiplex immunoassay from PBMCs. IFN-γ measurement was unsuccessful from 1 6wk-sample, 1 3mo-sample, and 3 6mo-samples stimulated with DMSO or S_wt. IL-2 measurement was unsuccessful from 3 6wk-samples, 5 3mo and 6mo-samples, and 3 negative samples stimulated with DMSO or S_wt. Data is represented as median and interquartile range. Statistical analysis was performed by Wilcoxon signed rank test for comparison of wt S peptide pool stimulations with Delta S peptide pool stimulations **(A, B)** and tetanus and SARS-CoV-2 wt and Delta S peptide pool stimulations with DMSO control **(B)**. Samples with no data on both data points were excluded from analysis. *p < 0.05; **p < 0.01; ***p < 0.001.

After stimulation with controls and peptide pools spanning SARS-CoV-2 wt and Delta spike proteins, interferon gamma (IFN-γ) and interleukin 2 (IL-2) mRNA levels in total cellular RNAs were measured with RT-qPCR from PBMC samples of 15 vaccinees, 10 COVID-19 patients, and 10 negative controls. Delta spike peptide pool stimulation was performed to 7/15 vaccinees, 6/10 COVID-19 patients, and 3/10 negative controls. The expression of IFN-γ and IL-2 mRNA was detected in 93% (14/15) and 100% (7/7) of vaccinees after wt and Delta spike peptide pool stimulation, respectively, and mRNA levels were higher compared to mRNA levels in PBMCs collected from negative donors (**Figure 4A**). There was no statistically significant difference in IFN-γ and IL-2 mRNA expression between stimulation with wt and Delta spike peptide pool. Additionally, INF-γ and IL-2 mRNA expression levels were similar between COVID-19 patients and vaccinated HCWs.

In addition to cellular cytokine mRNA quantitation, the levels of secreted cytokines related to CD4^+^ and CD8^+^ activation (IFN-γ, IL-2, TNF-α, and perforin) were measured from stimulated PBMC supernatants. Stimulation of PBMCs of vaccinated individuals with wt and Delta spike peptide pools resulted in statistically significant increase in secretion of IFN-γ and IL-2 proteins compared to DMSO-control (p<0.01, **Figure 4B**). The levels of IFNγ and IL-2 were higher in vaccinees and patients compared to negative controls, which was in line with the observed increase in mRNA expression (**Figure 4A**). IFN-γ and IL-2 production remained at similar levels 6 weeks and 6 months after the vaccination and stimulation with wt or Delta peptide pools was equally efficient (p>0.05). The production of TNF-α and perforin were not upregulated in vaccinee or patient PBMCs compared to unvaccinated healthy controls (**Supplementary Figure 1**). However, reliable analysis of TNFα results was not possible since the measurement of TNFα was unsuccessful in 6/15 vaccinees, 1/10 patients, and 5/10 negative controls due to an unexplained aggregation of Luminex beads.

**Figure 5:**
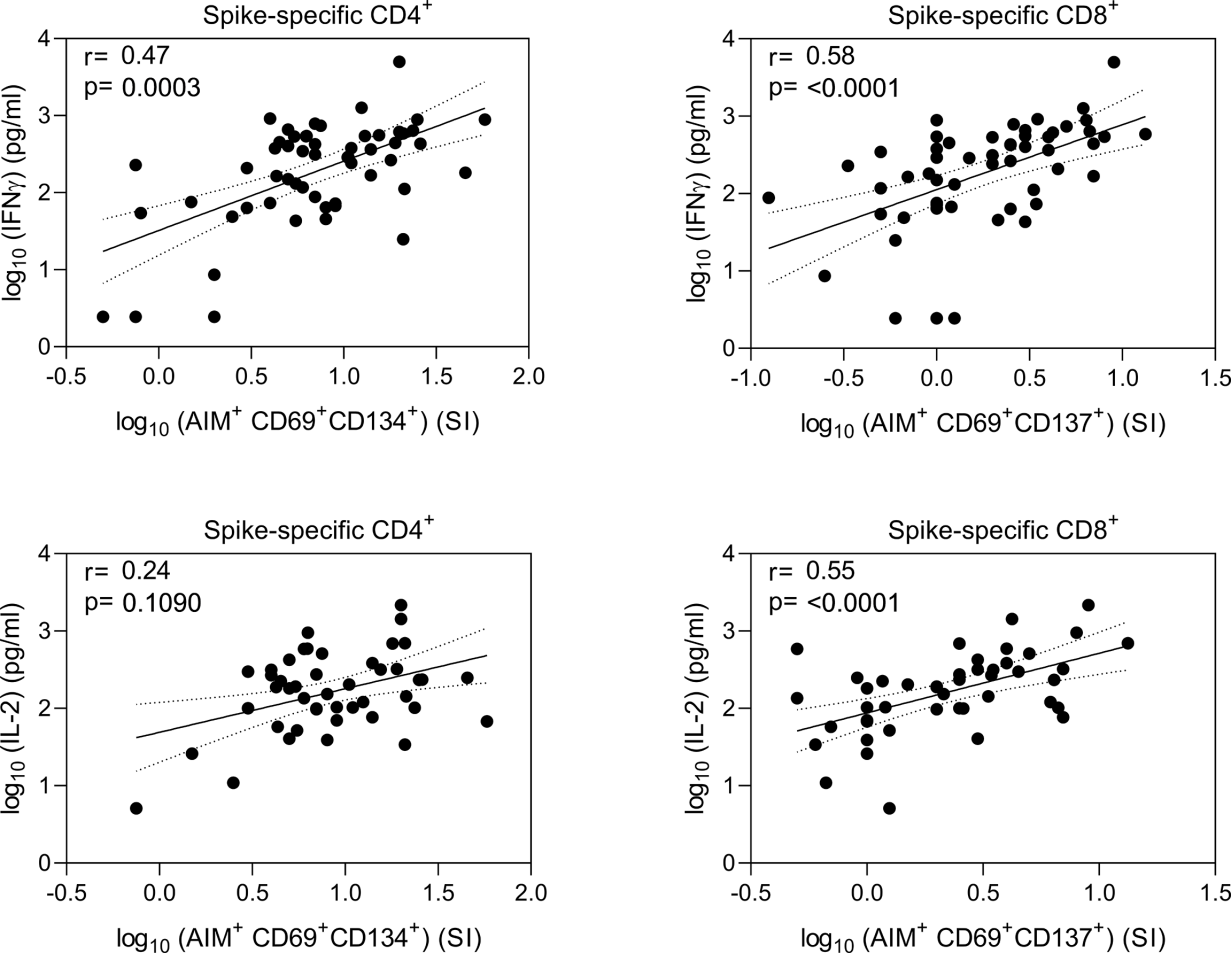
Correlation of spike-specific T cell responses. Nonparametric Spearman correlation analysis of secreted IFN-γ and IL-2 concentrations with AIM^+^ CD4^+^ and CD8^+^ T cells in wild type (wt) SARS-CoV-2 spike protein-specific peptide pool stimulated PBMCs. Results are shown from 15 BNT162b2 vaccinated health care workers at three timepoints after two doses of BNT162b2 vaccine (n=37 fro IFNg and 32 for IL-2), 10 COVID-19 patients, and 8 for IFNG and 3 for IL-2 healthy non-vaccinated controls. Dotted lines indicate 95% CI.

Correlation analysis of IFN-γ and IL-2 protein concentrations with the expression of T cell activation markers revealed high correlation between T cell activation and production of Th1 type cytokines (**Figure 5**). Activated CD8^+^ T cells had higher correlation with secretion of IFN-γ and IL-2 proteins (r=0.58 and r=0.55, respectively, p<0.0001) compared to CD4^+^ T cells. Activated CD4^+^ T cells correlated moderately with the levels of IFN-γ (r=0.47, p<0.001), while correlation with IL-2 was low and statistically non-significant (r=0.24, p=0.1090).

### Comparison of Humoral Immune Responses to Cell-Mediated Responses

**Figure 6:**
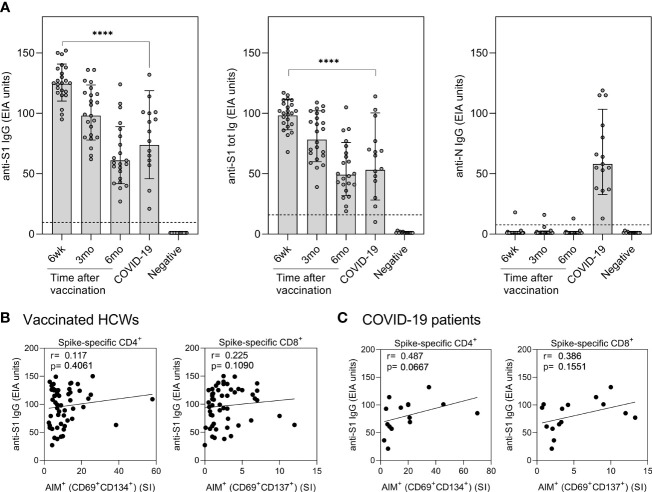
Correlation of humoral immunity to cell-mediated immunity after BNT162b2 vaccination n = 23 and SARS-CoV-2 infection. **(A)** Anti-SARS-CoV-2 S1-specific IgG, S1 total Ig, and N protein-specific IgG antibody responses were measured from samples collected at 6 weeks, 3 months, and 6 months after vaccination n = 23 or 1 month after PCR confirmed SARS-CoV-2 infection (n=15). Serum antibody levels of vaccinated and infected individuals were compared with negative controls (n=13) that had not received any COVID-19 vaccines or suffered from a previous SARS-CoV-2 infection. Bars represent the geometric mean titers. The cut-off values for a positive test result are shown with dotted line Statistical analysis was performed with Mann Whitney U-test for comparison of 6wk vaccinee samples with COVID-19 patient samples. ****p<0.0001. **(B,C)** The correlation of anti-SARS-CoV-2 S1 IgG antibody levels with SARS-CoV-2 (wt) spike specific CD4^+^ and CD8^+^ T cell responses in **(B)** vaccinated HCWs (samples collected 6 week, 3 months, and 6 months after vaccination, n= 52) and **(C)** COVID-19 patients (samples collected 1 month post onset of symptoms, n=15). Correlation was analyzed with the Spearman’s correlation and Spearman’s r is indicated in the figures.

In addition to analyzing SARS-CoV-2 spike-specific T cell immunity, spike and nucleocapsid-specific antibody responses induced by COVID-19 mRNA vaccination or COVID-19 disease were measured. Six weeks after the first vaccine dose (three weeks after the second dose) all vaccinees produced high levels of anti-S1 IgG and total Ig (IgG, IgA, and IgM) antibodies, and the mean anti-S1 antibody levels were significantly higher compared to antibody levels in hospitalized COVID-19 patients (p<0.0001) (**Figure 6A**). Vaccine-induced spike-specific antibody levels decreased gradually, and six months after the vaccination S1 protein-specific antibody levels were reduced more than two-fold. However, the exact fold-decrease could not be confirmed since some 6wk samples were saturated with used serum dilutions. All hospitalized COVID-19 patients were seropositive for SARS-CoV-2 N-protein specific IgG antibodies, whereas only one of the vaccinated HCWs had anti-N IgG antibodies (**Figure 6A**). The HCW who was positive for anti-N IgG antibodies had been anti-SARS-CoV-2 N antibody positive but anti-S1 antibody negative before vaccination (11, 13) and thus the persisting low levels of anti-N antibodies were considered to be induced by a previous seasonal coronavirus infection.

The correlation between SARS-CoV-2 S1-specific antibody levels and spike-specific CD4^+^ and CD8^+^ T cell stimulatory indices was analyzed for vaccinated HCWs and COVID-19 patients (**Figures 6B, C**). To increase the number of variables, all samples from the vaccinees collected at six weeks, three months, and six months after vaccination were combined in the calculations. The levels of S1-specific antibodies in vaccinees did not correlate with CD4^+^ or CD8^+^ T cell responses, whereas in COVID-19 patients anti-S1 IgG antibody levels had a trend with CD4^+^ (r=0.487, p=0.0667) and CD8+ (r=0.386, p=0.1551) T-cell responses. Only S1-specific antibodies correlated with spike-specific T cell responses, while N-specific antibodies from patient samples did not show any correlation with S-specific stimulatory indices (r= -0.012 for CD4^+^ and r= -0.036 for CD8^+^).

## Discussion

The COVID-19 pandemic has now continued for more than two years. The development of effective vaccines and disease control strategies relies on accurate data on the dynamics of vaccine-induced immune responses against emerging variants of SARS-CoV-2. Although previous studies have demonstrated declining neutralizing antibody levels against SARS-CoV-2 variants with immune-escape mutations (14), epidemiological data suggest high vaccine efficacy against symptomatic (15) or severe disease and hospital admissions (16) indicating immune protection by cellular mechanisms. In this study, we demonstrate an efficient activation of SARS-CoV-2 spike-specific CD4^+^ and CD8^+^ T cells after BNT162b2 vaccination and after acute COVID-19 infection and persistence of robust T cell responses at least for six months after vaccination.

The BNT162b2 mRNA vaccine explored in the present study is one of the most frequently used COVID-19 vaccines in Finland and in the European Union (17, 18). After BNT162b2 vaccination, CD4^+^ T cell responses directed against spike-protein derived peptides were detected in all vaccinees, while CD8^+^ T cell responses were detected in ca. 70% of the vaccinees. We found no statistically significant differences in the overall T cell responses between vaccinated and convalescent individuals. However, we focused only on spike-specific responses although COVID-19 patients could also have T cell responses against other structural and non-structural SARS-CoV-2 proteins (19, 20). Our data on cytokine expressions suggest that CD4^+^ T cell responses are well sustained and directed towards Th1 type response: we found clearly increased IFN-γ and IL-2 mRNA expression followed by efficient secretion of these cytokines. Increased gene expression of IL-4 was not detected. In contrast to some other studies (21), we did not detect increased production of TNF-α or granzyme B, indicating that CD8^+^ T cells were too infrequent or less functional after stimulation with SARS-CoV-2 spike protein peptide pools.

It has been somewhat difficult to study CD8+ T cell responses since the number of activated CD8+ T cells in blood has been low likely because CD8+ cells tend to be distributed all around the body following infection (22) limiting the amount of circulating memory CD8^+^ cells (23). However, the number of circulating CD8^+^ memory T cells appears not to be directly proportional to the number of tissue-resident memory CD8+ cells mediating protection (21, 22, 24, 25). This may further complicate the interpretation of the correlation of CD8^+^ T cell results obtained from PBMCs to the cell-mediated protection against COVID-19. However, our data revealed long-lasting spike-protein targeted CD4^+^ and CD8^+^ responses in the majority of samples both from vaccinees and convalescent phase patients.

During our study period in summer and autumn 2021, the local circulating variant was Delta (B.1.617.2) (26) and even though Delta was to a great extent superseded by Omicron (B.1.1.529) in December 2021, we focused on analyzing the T cell responses against four VOCs; Alpha (B.1.1.7), Beta (B.1.351), Gamma (P.1), and Delta. Consistent with previous studies (10), our data strongly emphasize that the amino acid changes in VOCs do not alter T cell activity and the T cell responses are maintained against all four variants for at least 6 months after the vaccination. Since the T cell responses to S protein specific peptides is robust, it is expected that individual amino acid changes in the present and future VOCs are unlikely to markedly impair efficient T cell responses. Furthermore, the functional properties of the T cells, characterized by the production of INF-γ and IL-2, were similar against wt and Delta variants, both in vaccinated individuals and convalescent phase patients.

Our study has some limitations. First, we used cryopreserved PBMCs which may lead to a loss of infrequent memory cells and decreased cell viability. Fortunately, however, freezing is not expected to impact final CD4/CD8 T cell ratios (27). On the other hand, using frozen cells enabled us to analyze all the samples from each individual simultaneously, which is an obvious advantage in terms of the reliability of the analyses. Second, the number of participants was rather low, and we did not analyze responses to other COVID-19 vaccines than BNT162b2. Third, our vaccinated HCWs were all at a working age, the study population was thus missing vaccinees over 60 years of age who could be more susceptible to infections due to weakening activation of T cells. Fourth, we optimized the AIM protocol using maximum of 48 hours stimulation time and did not test for longer incubation times such as 72 hours or longer. Fifth, we used longer 15-mer peptide pools for each stimulation, which could underestimate the available SARS-CoV-2 specific CD8+ cells, since 9-to 10-mer peptides are more ideal for HLA class 1 binding (28).

Even though emerging SARS-CoV-2 variants can evade recognition by neutralizing antibodies - especially when the level of antibodies declines over time, cell-mediated immune responses are much better sustained. Since SARS-CoV-2 S-protein targeted cell-mediated immunity is clearly more long-lasting and robust than the humoral immunity, it most probably contributes remarkably to the long-term protection against the SARS-CoV-2 variants as well. Future analyses should cover humoral and cell-mediated immunity using different vaccine combinations and those after the third vaccine dose. Furthermore, it will be crucial to investigate the persistence of COVID-19 vaccine induced immunity to the present and future variants of concern.

## Data Availability Statement

The raw data supporting the conclusions of this article will be made available by the authors, without undue reservation.

## Ethics Statement

The studies involving human participants were reviewed and approved by The Ethics Committees of the Southwest Finland health district and the Helsinki-Uusimaa health district. The patients/participants provided their written informed consent to participate in this study.

## Author Contributions

AH, PJ, AHä, AK, LK, JL and IJ designed the experiments. AH, PJ and JH performed the experiments. AH, PJ, JH, IJ and LK analyzed the data. OL, SV, MM, JT, QH, SP, AH, JO, TV, PAT and LI contributed to the data collection and data design. AH, PJ, LK and IJ wrote the manuscript and all authors revised and approved the manuscript for publication. All authors contributed to the article and approved the submitted version.

## Funding

This work was supported by Jane and Aatos Erkko Foundation (grant numbers 3067-84b53 and 5360-cc2fc to IJ). The Academy of Finland (grant numbers 336410 and 337530 to IJ, and 336439 and 335527 to AK). Sigrid Jusélius Foundation (to IJ and LK). the Finnish Medical Foundation (to AK). and The Turku University Hospital Research Foundation (to PAT, LI, and JL).

## Conflict of Interest

The authors declare that the research was conducted in the absence of any commercial or financial relationships that could be construed as a potential conflict of interest.

## Publisher’s Note

All claims expressed in this article are solely those of the authors and do not necessarily represent those of their affiliated organizations, or those of the publisher, the editors and the reviewers. Any product that may be evaluated in this article, or claim that may be made by its manufacturer, is not guaranteed or endorsed by the publisher.
