# Supplementary material for: Long-Lasting T Cell Responses in BNT162b2 COVID-19 mRNA Vaccinees and COVID-19 Convalescent Patients
Source: Front Immunol. 2022 Apr 22;13:869990. doi: 10.3389/fimmu.2022.869990 (PMC9073085; doi:10.3389/fimmu.2022.869990)
Supplement: Supplementary file 1 [file DataSheet_1.pdf]

Supplementary table 1. Peptide pools mapping SARS-CoV-2 spike protein.

Product	Manufacturer	Cat#	Number of spike aa changes [a]
PepMix™ SARS-CoV-2 (Spike Glycoprotein)	JPT Peptide Technologies	PM-WCPV-S-1	-
PepMix™ SARS-CoV-2 (Spike B.1.1.7 / Alpha)	JPT Peptide Technologies	PM-SARS2-SMUT01-1	10
PepMix™ SARS-CoV-2 (Spike B.1.351 / Beta)	JPT Peptide Technologies	PM-SARS2-SMUT02-1	10
PepMix™ SARS-CoV-2 (Spike P.1 / Gamma)	JPT Peptide Technologies	PM-SARS2-SMUT03-1	12
PepMix™ SARS-CoV-2 (Spike B.1.617.2 / Delta)	JPT Peptide Technologies	PM-SARS2-SMUT06-1	10

a, compared to spike protein of wild type SARS-CoV-2

Supplementary table 2. Fluorochrome labelled antibodies in FACS.

Antibody	Fluorochrome	Manufacturer	Cat#
Anti-human CD45	APC-eFluor780	Invitrogen/Life technologies	47-0459-42
Anti-human CD3	eFluor506	Invitrogen/Life technologies	69-0038-42
Anti-human CD4	eFluor450	Invitrogen/Life technologies	48-0049-42
Anti-human CD8a	PerCP-eFluor710	Invitrogen/Life technologies	46-0087-42
Anti-human CD69	PE	BD Biosciences	555531
Anti-human CD134	PE/Cyanine7	BioLegend	350012
Anti-human CD137	APC	BioLegend	309810

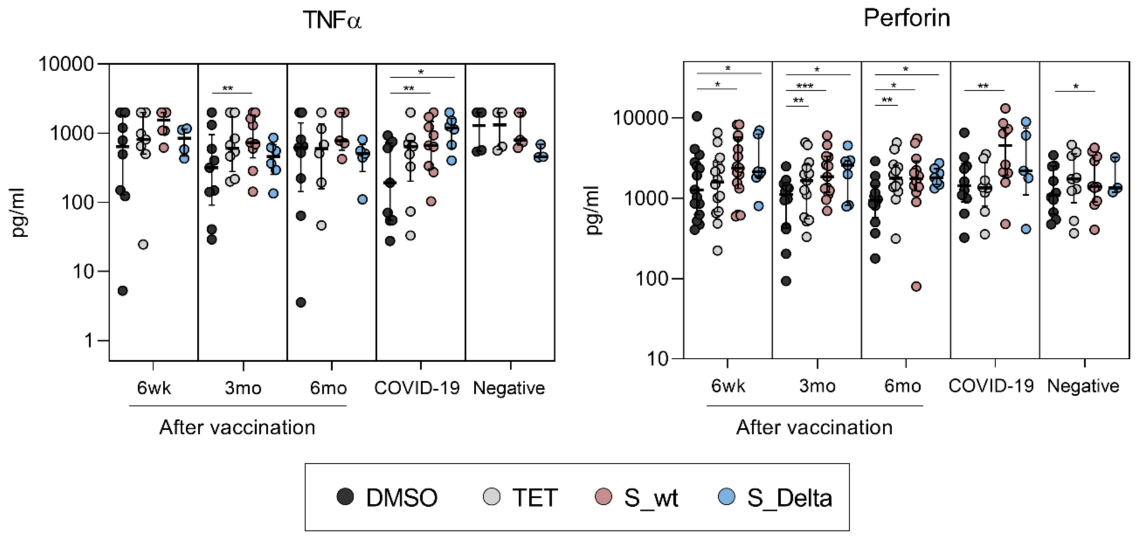

Supplementary Fig. 1 Measurement of secreted TNFα and perforin after T cell stimulation. PBMCs collected from vaccinated HCWs (n=15), COVID-19 patients (n=10), and negative controls (n=10) were stimulated with DMSO, tetanus toxoid (TET), and SARS-CoV-2 peptide pools spanning the spike protein of wild type Wuhan Hu (S_wt) and Delta variant (S_Delta). Secreted TNFα and perforin levels were measured with Luminex based multiplex immunoassay. Delta variant S peptide stimulation was performed to 7/15 vaccinated, 6/10 COVID-19 patients, and 3/10 negative controls. Data is represented as median and interquartile range. Statistical analysis was performed with Wilcoxon signed rank test for comparison of S_wt stimulations with S_Delta stimulations, and comparison of tetanus and S_wt and S_Delta stimulations with negative DMSO control. Samples with no data on both data points were excluded from statistical analysis. $*p<0.05$; $**p<0.01$; $***p<0.001$.
